# Long-Term Feeding of Chitosan Ameliorates Glucose and Lipid Metabolism in a High-Fructose-Diet-Impaired Rat Model of Glucose Tolerance

**DOI:** 10.3390/md13127067

**Published:** 2015-12-10

**Authors:** Shing-Hwa Liu, Fang-Ying Cai, Meng-Tsan Chiang

**Affiliations:** 1 Institute of Toxicology, College of Medicine, National Taiwan University, Taipei 100, Taiwan; shinghwaliu@ntu.edu.tw; 2 Department of Medical Research, China Medical University Hospital, China Medical University, Taichung 104, Taiwan; 3 Department of Food Science, National Taiwan Ocean University, Keelung 202, Taiwan; arer77@yahoo.com.tw

**Keywords:** chitosan, fructose, plasma lipids

## Abstract

This study was designed to investigate the effects of long-term feeding of chitosan on plasma glucose and lipids in rats fed a high-fructose (HF) diet (63.1%). Male Sprague-Dawley rats aged seven weeks were used as experimental animals. Rats were divided into three groups: (1) normal group (normal); (2) HF group; (3) chitosan + HF group (HF + C). The rats were fed the experimental diets and drinking water *ad libitum* for 21 weeks. The results showed that chitosan (average molecular weight was about 3.8 × 10^5^ Dalton and degree of deacetylation was about 89.8%) significantly decreased body weight, paraepididymal fat mass, and retroperitoneal fat mass weight, but elevated the lipolysis rate in retroperitoneal fats of HF diet-fed rats. Supplementation of chitosan causes a decrease in plasma insulin, tumor necrosis factor (TNF)-α, Interleukin (IL)-6, and leptin, and an increase in plasma adiponectin. The HF diet increased hepatic lipids. However, intake of chitosan reduced the accumulation of hepatic lipids, including total cholesterol (TC) and triglyceride (TG) contents. In addition, chitosan elevated the excretion of fecal lipids in HF diet-fed rats. Furthermore, chitosan significantly decreased plasma TC, low-density lipoprotein cholesterol (LDL-C), very-low-density lipoprotein cholesterol (VLDL-C), the TC/high-density lipoprotein cholesterol (HDL-C) ratio, and increased the HDL-C/(LDL-C + VLDL-C) ratio, but elevated the plasma TG and free fatty acids concentrations in HF diet-fed rats. Plasma angiopoietin-like 4 (ANGPTL4) protein expression was not affected by the HF diet, but it was significantly increased in chitosan-supplemented, HF-diet-fed rats. The high-fructose diet induced an increase in plasma glucose and impaired glucose tolerance, but chitosan supplementation decreased plasma glucose and improved impairment of glucose tolerance and insulin tolerance. Taken together, these results indicate that supplementation with chitosan can improve the impairment of glucose and lipid metabolism in a HF-diet-fed rat model.

## 1. Introduction

Obesity is a worldwide health care issue and is known to increase the risk of chronic diseases, such as diabetes, hypertension, and cardiovascular disorders [1,2]. The food industry uses high quantities of fructose as a sweetener in candy, chocolate, and beverages, which leads to fructose consumption increasing rapidly from 37 g/day in 1970 [3] to 49 g/day in 2004 [4]. However, many studies have shown that a high-fructose (HF) diet can produce hypertriglyceridemia, oxidative stress, obesity, and insulin resistance due to high hepatic lipogenesis, high very-low-density lipoprotein (VLDL)-triglyceride (TG) secretion, low fat oxidation, and low insulin receptor mRNA expression in the skeletal muscle and liver [5,6]. It has been indicated that high fructose intake is increasingly recognized as causative in the development of prediabetes and metabolic syndrome [7].

Chitosan, a biopolymer of glucosamine derived from chitin, which is chemically similar to cellulose, is not digestible by mammalian digestive enzymes and has been widely employed as a dietary supplement [8]. Chitosan has been shown to reduce liver cholesterol by decreasing cholesterol absorption and increasing bile acid and fat excretions in cholesterol-fed rats and hamster [9,10]. In addition, several animal studies have shown that chitosan possesses antidiabetic potential for type 1 and 2 diabetes [11,12,13]. Yao *et al*. [14] found high-molecular-weight (MW) chitosan significantly decreased plasma glucose and plasma total cholesterol (TC) and increased high-density lipoprotein cholesterol (HDL-C) and fecal cholesterol excretion in streptozotocin-induced diabetic rats. Consumption of chitosan has a beneficial effect by reducing body weight, TC and low-density lipoprotein cholesterol (LDL-C), and glucose, and increasing cholesterol excretion [15,16]. Moreover, several clinical studies have shown the beneficial effects of chitosan and other nutraceuticals on lipid-lowering treatment [17,18]. Therefore, it is possible that the hypolipidemic and hypoglycemic effects of chitosan may come to be recognized as a promising therapeutic strategy for metabolic syndrome and prediabetes. In the present study, we aim to investigate the effects and possible mechanisms of long-term feeding of high-MW chitosan on lipid responses and lipid-related metabolic changes in rats with high-fructose-diet-impaired glucose tolerance.

## 2. Results

### 2.1. Effects of Chitosan on Body Weight and Tissue Weight in HF-Diet-Fed Rats

**Figure 1:**
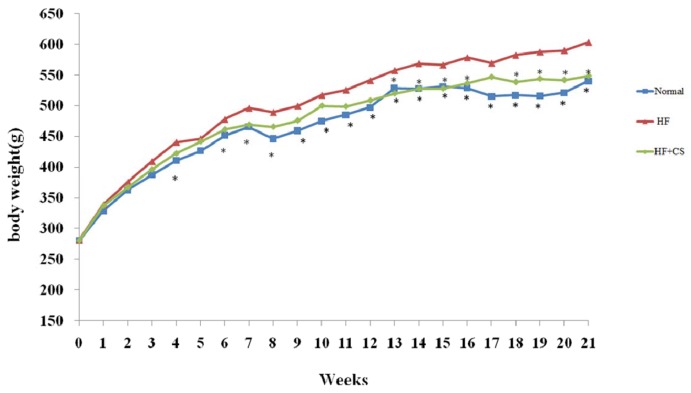
The changes of body weight in normal and fructose rats fed different experimental diets for 21 weeks in prophylactic experiment. Results are expressed as mean ± SD for *n* = 8 rats per group. Normal: normal control, HF: high fructose, HF + CS: high-fructose + chitosan (prophylactic experiment) * *p* < 0.05 compared with H by independent-samples *t*-test.

The rats fed an HF diet for 21 weeks had increased body weight (Figure 1), liver weight, and adipose weight (Table 1). However, chitosan supplementation reverses the increased body weight, adipose weight, and liver weights. The food intake and urine volume in rats fed with a high-fructose diet, with or without supplementation of chitosan, for 21 weeks were not altered (Table 1) (*p* > 0.05).

**Table 1:** The changes of liver weight and adipose tissue weight in normal and high-fructose rats fed the different experimental diets.

Tissue Weight	Groups		
	Normal	HF	HF + CS
Liver weight (g)	28.1 ± 4.1 *	35.7 ± 6.3	23.7 ± 5.2 *
Relative liver weight (g/100 g BW)	5.3 ± 0.6 *	6.1 ± 0.9	4.4 ± 0.7 *
Adipose tissue weight(g)	21.4 ± 5.1 *	28.6 ± 8.4	19.2 ± 5.6 *
Relative adipose tissue weight (g/100 g BW)	4.0 ± 1.0	4.9 ± 1.2	3.6 ± 1.1 *
Retroperitoneal adipose weight(g)	13.9 ± 3.3 *	18.7 ± 5.7	12.5 ± 4.0 *
Relative retroperitoneal adipose weight (g/100 g BW)	2.6 ± 0.6	3.2 ± 0.8	2.4 ± 0.8 *
Epididymal adipose weight(g)	7.5 ± 2.3	9.9 ± 2.9	7.1 ± 2.2 *
Relative epididymal adipose weight (g/100 g BW)	1.4 ± 0.4	1.7 ± 0.5	1.3± 0.4 *

Results are expressed as mean ± SD for *n* = 8 rats per each group. Normal: normal control, HF: high fructose, HF + CS: high fructose + chitosan. * *p* < 0.05 compared with HF by independent-sample’s *t*-test.

### 2.2. Effects of Chitosan on Plasma Glucose and Lipid Metabolism in HF-Diet-Fed Rats

**Figure 2:**
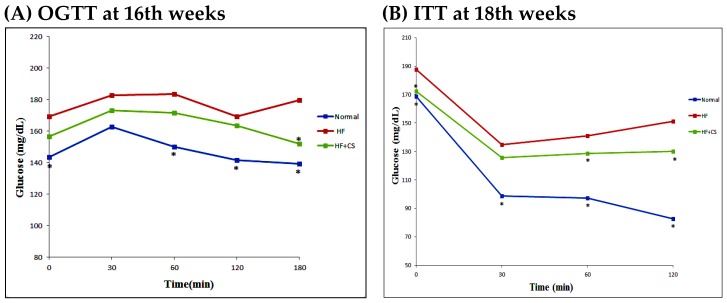
The changes of plasma glucose concentration after oral glucose tolerance test (OGTT) (**A**) and insulin tolerance test (ITT) (**B**) performed in rats fed the different experimental diets for 16 and 18 weeks, respectively. Results are expressed as mean ± SD for *n* = 8 rats per each group. Normal: normal control; HF: high fructose; HF + CS: high fructose + chitosan; * *p* < 0.05 compared with H by independent-samples *t*-test.

**Figure 3:**
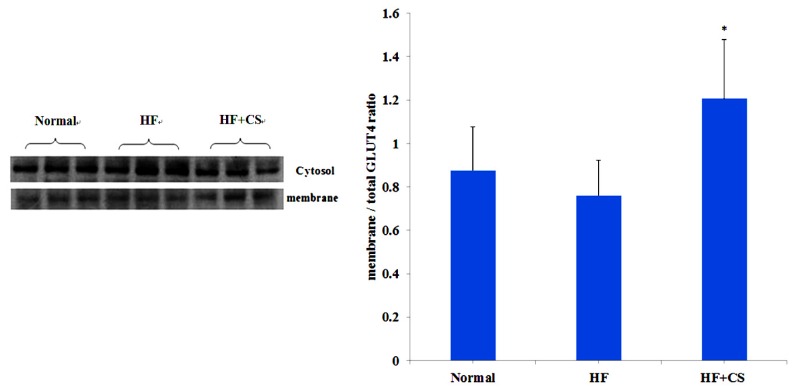
The change of soleus muscle glucose transporter 4 (GLUT4) in normal and high-fructose rats fed the different experimental diets. Results are expressed as mean ± SD for *n* = 8 rats per each group. Normal: normal control, HF : high fructose, HF + CS: high fructose + chitosan; * *p* < 0.05 compared with H by independent-samples *t*-test.

**Figure 4:**
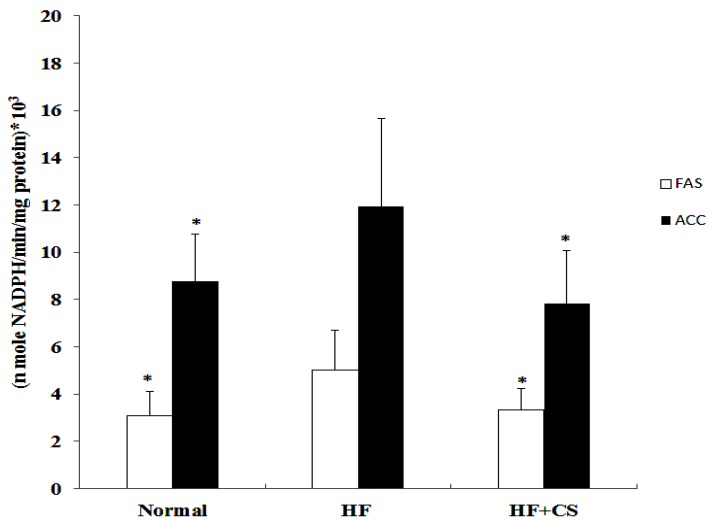
The effect of hepatic Fatty acid synthase (FAS) and Acyl-CoA crboxylase (ACC) in normal and high-fructose rats fed the different experimental diets. Results are expressed as mean ± SD for *n* = 8 rats per each group. Normal: normal control, HF : high fructose, HF + CS: high fructose + chitosan * *p* < 0.05 compared with H by independent-samples *t*-test.

The level of fasting plasma glucose in high-fructose-diet-fed rats was significantly increased, but supplementation of chitosan did not change the plasma glucose (Table 2). The plasma insulin levels in HF-diet-fed rats supplemented with chitosan were significantly lower than the HF-diet-fed without chitosan. A high-fructose diet significantly increased plasma glucose at 60, 120, and 180 min after OGTT performance, and at 30, 60, and 120 min after ITT performance, indicating that a high-fructose diet induces an insulin-resistant state. However, chitosan reversed the increased plasma glucose at 180 min for OGTT and at 60 and 120 min for ITT (Figure 2). Moreover, GLUT4 translocation evaluated by the difference in cytosol and membrane GLUT4 protein levels in the soleus muscle was not affected by fructose diet feeding (Figure 3). Dietary supplementation with chitosan significantly increased GLUT4 translocation in HF-diet-fed rat soleus muscles. These results could reflect the amelioration of insulin resistance by supplementation of chitosan in HF-diet-fed rats. On the other hand, HF-diet-fed rats supplemented with chitosan had lower plasma levels of TC, LDL-C + VLDL-C, and TC/HDL-C ratio, but the plasma TG and free fatty acid levels in HF-diet-fed rats were enhanced by the supplementation of chitosan in the diet (Table 2). The liver fatty acid synthase and acetyl CoA carboxylase activities were increased in HF-diet-fed rats, but chitosan supplementation reverses the increase in these activities (Figure 4). The decreased leptin, IL-6, and TNF-α, and increased adiponectin were observed after chitosan treatment when compared to the HF-alone group (Table 3). In this study, high fructose increased the total cholesterol and triglyceride contents of the liver. However, rats fed a chitosan diet displayed decreased cholesterol and triglyceride contents in the liver (Table 4). These results indicated that chitosan feeding can reduce hepatic accumulation in rats fed a diet enriched in fructose. On the other hand, chitosan supplementation increased fecal cholesterol and triglyceride in rats fed a high-fructose diet (Table 5), indicating that the reduced hepatic lipid accumulation may be related to the increased fecal lipids.

**Table 2:** The changes of plasma glucose, insulin, and HOMA-IR and plasma lipids in normal and high-fructose rats fed the different experimental diets.

Parameters	Groups		
	Normal	HF	HF + CS
Glucose (mg/dL)	187.0 ± 7.6 *	215.9 ± 32.6	191.3 ± 19.1
Insulin (μg/L)	0.9 ± 0.3	0.8 ± 0.2	0.6 ± 0.2 *
Total cholesterol (mg/dL)	193.4 ± 31.0 *	288.2 ± 110.5	177.4 ± 34.6 *
HDL-C (mg/dL)	53.6 ± 8.3	51.2 ± 17.4	63.6 ± 17.8
LDL-C + VLDL-C (mg/dL)	147.4 ± 35.6 *	231.8 ± 102.1	113.8 ± 26.2 *
TC/HDL-C	3.9 ± 0.9 *	5.1 ± 1.3	3.0 ± 0.3 *
HDL-C/(LDL-C + VLDL-C)	0.4 ± 0.2	0.3 ± 0.1	0.5 ± 0.1 *
Triglyceride (mg/dL)	60.9 ± 11.4	65.0 ± 6.0	92.2 ± 26.0 *

Results are expressed as mean ± SD for *n* = 8 rats per each group. Normal: normal control, HF: high fructose, HF + CS: high fructose + chitosan. * *p* < 0.05 compared with HF by independent-samples *t*-test.

**Table 3:** The changes of plasma TNF-α, IL-6, adiponectin, and leptin concentrations in normal and high-fructose rats fed the different experimental diets.

Parameters	Groups		
	Normal	HF	HF + CS
TNF-α (pg/mL)	16.4 ± 1.0 *	20.6 ± 4.4	15.0 ± 4.2 *
IL-6 (pg/mL)	19.3 ± 1.7 *	23.3 ± 2.9	20.4 ± 2.9 *
Adiponectin (μg/mL)	16.5 ± 2.5 *	8.00 ± 2.8	11.5 ± 3.1 *
Leptin (ng/mL)	6.5 ± 2.0	9.8 ± 3.5	4.68 ± 1.5 *
Free fatty acids	1.1 ±0.3	1.1 ±0.2	1.4 ±0.3 *

Results are expressed as mean ± SD for *n* = 8 rats per each group. Normal: normal control, HF: high fructose, HF + CS: high fructose + chitosan. * *p* < 0.05 compared with HF by independent-samples *t*-test.

**Table 4:** The changes of hepatic lipid concentrations in normal and high-fructose rats fed the different experimental diets.

Hepatic Lipids	Groups		
	Normal	HF	HF + CS
**Total Cholesterol**			
(mg/g liver)	83.6 ± 14.5 *	121.0 ± 19.3	62.5 ± 17.0 *
(g/liver)	2.4 ± 0.5 *	4.3 ± 1.0	1.1 ± 0.4 *
**Triglyceride**			
(mg/g liver)	43.7 ± 6.8 *	57.9 ± 16.0	30.4 ± 11. *
(g/liver)	1.2 ± 0.3 *	2.0 ± 0.6	0.8 ± 0.2 *

Results are expressed as mean ± SD for *n* = 8 rats per each group. Normal: normal control, HF: high fructose, HF + CS: high fructose + chitosan. * *p* < 0.05 compared with HF by independent-samples *t*-test.

**Table 5:** The changes of fecal weight and fecal lipids in normal and high-fructose rats fed the different experimental diets.

Fecal Weight and Lipids	Groups		
	Normal	HF	HF + CS
Feces wet weight (g/day)	2.2 ± 0.3	2.3 ± 0.3	2.4 ± 0.4
Feces dry weight (g/day)	1.9 ± 0.3	2.0 ± 0.3	1.9 ± 0.2
**Total Cholesterol**			
(mg/g feces)	5.2 ± 2.0	5.4 ± 1.3	16.5 ± 3.0 *
(mg/day)	10.2 ± 3.5	10.7 ± 2.3	30.1 ± 4.6 *
**Triglyceride**			
(mg/g feces)	1.7 ± 0.4	1.6 ± 0.3	3.4 ± 0.6 *
(mg/day)	3.1 ± 0.9	3.2 ± 0.6	6.3 ± 1.4 *

Results are expressed as mean ± SD for *n* = 8 rats per each group. Normal: normal control, HF: high fructose, HF + CS: high fructose + chitosan. * *p* < 0.05 compared with HF by independent-samples *t*-test.

### 2.3. Effects of Chitosan on the ANGPTL4 Protein Expression

**Figure 5:**
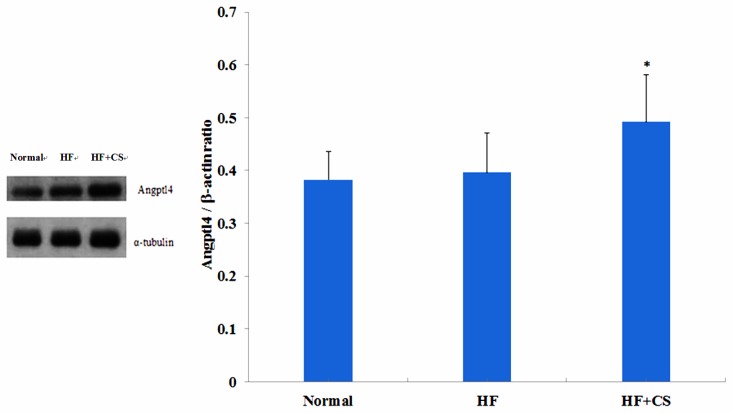
The change of plasma Angptl4 /α-tubulin ratio in normal and high-fructose rats fed the different experimental diets. Results are expressed as mean ± SD for *n* = 8 rats per each group. Normal: normal control, HF : high fructose, HF + CS: high fructose + chitosan. * p < 0.05 compared with HF by independent-sample’s *t*-test.

Plasma ANGPTL4 protein expression was not affected in HF-diet-fed rats but was significantly increased in chitosan-supplemented, high-fructose-diet-fed rats (Figure 5).

### 2.4. Effects of Chitosan on the Hepatic Enzyme Activities and Lipolysis

**Figure 6:**
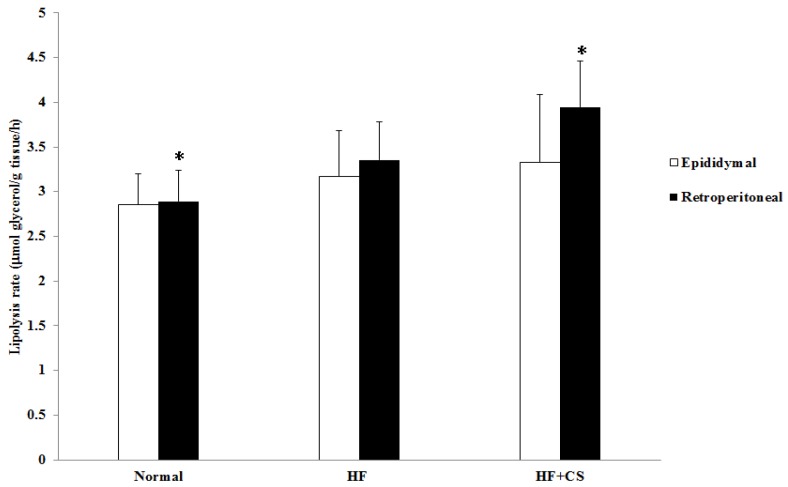
The changes of lipolysis rate of retroperitoneal and epididymal fat pads in normal and high fructose rats fed the different experimental diets. Results are expressed as mean ± SD for *n* = 8 rats per each group. Normal: normal control, HF: high fructose, HF+CS: high fructose + chitosan * *p* < 0.05 compared with HF by independent-samples *t*-test.

The increased hepatic enzymes of lipid biosynthesis (acetyl CoA carboxylase and fatty acid synthase) in HF-diet-fed rats could be efficiently ameliorated by chitosan supplementation (Figure 4). It is interesting to note that a high-fructose diet decreased the lipolysis rate, but supplementation of chitosan in the diet significantly elevated the lipolysis rate in retroperitoneal fats of high fructose-diet-fed rats (Figure 6).

## 3. Discussion

High-fructose diet (HFD) intake induced hypertrophic visceral adipose tissue [19]. In addition, Catena *et al.* [6] found that HFD leads to insulin resistance in rats by decreasing insulin receptor mRNA expression in the skeletal muscle and liver. In the present study, we found that long-term HF-diet feeding produces impairment of glucose and lipid metabolism in rats, including the induction of hyperglycemia, insulin resistance, and hyperlipidemia. Therefore, this HF-diet-fed animal model shows symptoms similar to type 2 diabetes. The present work further showed that supplementation of chitosan in the diet can mitigate the increased body weight, hypercholesterolemia, and increased insulin resistance induced by HF-diet feeding in rats. These results indicate that chitosan possesses the ability to improve the impairment of glucose and lipid metabolism in a HF-diet-fed rat model.

We have reported that chitosan can activate AMPK phosphorylation in a high-fat-fed rat model [20] and in a high-sucrose-fed rat model [21]. In addition, Liu *et al.* [11] have demonstrated that chitosan can activate AMPK phosphorylation and reverse the increase in liver PECK and phospho-38 protein expressions, and reverse the decrease in skeletal AKT protein phosphorylation and GLUT4 translocation in diabetic rats, indicating that chitosan ameliorates hyperglycemia through a decrease in liver gluconeogenesis and an increase in skeletal muscle glucose use. In the present study, chitosan supplementation decreased plasma glucose, increased GLUT4 translocation, and improved oral glucose tolerance test and insulin tolerance test results, suggesting that the hyperglycemia and insulin resistance induced by a long-term high-fructose diet can be alleviated by chitosan; this effect is possibly through the activation of AMPK phosphorylation in an HFD-fed rat model.

Adipose tissue is an important endocrine organ that secretes multiple adipocytokines affecting energy metabolism and insulin sensitivity [22,23]. Hypertrophic adipocytes may increase plasma leptin, TNF-α, and IL-6 concentrations but decrease the plasma adiponectin level. Results from this study show that chitosan feeding significantly reduced adipose tissue weight (paraepididymal and retroperitoneal). Plasma TNF-α and IL-6 concentrations were increased and adiponectin was decreased in an HF-fed-diet rat model. Consistent with previous results [12], our results showed that chitosan feeding could reverse the increased TNF-α and IL-6 and the decreased adiponectin levels in an HF-fed-diet rat model. These results indicate that chitosan may regulate plasma adipocytokines by lowering adipose tissue weight in an HFD-rat model.

Hormone-sensitive lipase (HSL) is an enzyme that promotes fatty tissue decomposition and conducts lipolysis from triacylglycerol to monoacylglycerol and free fatty acids [24]. A high-fructose diet may dysregulate the lipolysis and lipid metabolism, decreasing the lipolysis rate in visceral adipocytes, which may promote adipocyte enlargement by facilitating TG accumulation in adipocytes [25]. Costabile *et al.* [26] showed that HSL activity is lower in type 2 diabetes, which may be a mechanism by which obesity is accelerated in diabetes. In the present study, HF-diet feeding decreases the lipolysis rate in retroperitoneal fats. Similar to the actions observed in the previous study [12], our results also showed that the rate of lipolysis in retroperitoneal adipose tissue was higher in the chitosan supplementation group. Therefore, chitosan feeding could increase the rate of lipolysis in adipose tissue, thereby reducing the adipose tissue weight in HFD rats.

Diabetic patients have abnormal lipid metabolism [27]. Supplementation of chitosan in the diet has been found to lower plasma TC, VLDL-C, and LDL-C levels in high-cholesterol-diet-fed hamsters by increasing the fecal excretion of cholesterol and bile acid [10]. A study has also shown that chitosan significantly reverses the increased plasma TC and LDL-C levels in cholesterol-enriched-diet-fed rats by upregulating the liver LDL receptor expression [28]. Yao *et al*. [14] have also found that supplementation of chitosan in the diet for seven weeks reduces the plasma glucose, TC, and free fatty acid levels in streptozotocin-induced diabetic rats. In these studies, plasma TG levels were not affected by chitosan. Similarly, the present study showed that supplementation of chitosan significantly decreased the increased plasma levels of TC, LDL-C + VLDLC, and TC/HDL-C ratio in HF-diet-fed rats, but the plasma TG levels were increased.

ANGPTL4, a lipoprotein lipase and hepatic lipase inhibitor, can decrease the dietary fatty acid uptake in the liver, and further impairs plasma TG clearance [29,30]. PPARα can upregulate hepatic ANGPTL4 expressions and plasma ANGPTL4 concentrations that act as a LPL activity inhibitor [31]. ANGPTL4-related LPL activity inhibition caused the inhibition of LPL-dependent VLDL lipolysis, leading to hypertriglyceridemia [32]. Our previous study demonstrated that chitosan feeding can increase the hepatic PPARα expression [12]. In the present study, we found that long-term supplementation of chitosan in the diet significantly enhanced the plasma free fatty acid and TG levels in HF-diet-fed rats. Supplementation of chitosan in the diet could also significantly increase the plasma ANGPTL4 protein expression in HF-diet-fed rats, but HF-diet feeding alone did not affect the ANGPTL4 protein expression. Taken together, these results suggest that chitosan supplementation increases ANGPTL4 expression, which may be upregulated by PPARα, and then enhances the lipolysis rate in retroperitoneal fat tissues, resulting in the increase of plasma free fatty acid levels. The increased ANGPTL4 expression could also change the plasma LPL activity, which induces a decrease in hydrolysis of TG-rich lipoproteins, causing the increase of plasma TG levels.

Many studies have provided evidence to produce natural therapeutic agents using chitosan and its oligosaccharides as lead compounds for prevention and treatment of diabetes or age-related diseases such as cancer and inflammatory diseases [33,34,35]. Low MW chitosan has been demonstrated to exhibit an antidiabetic effect on genetically obese diabetic KK-Ay mice [13] and streptozotocin-induced diabetic rats [11]. High MW chitosan supplementation has been found to improve the impairment of lipid metabolism in high-sucrose-diet-fed rats [21] and streptozotocin-induced diabetic rats [14]. Anraku *et al.* [36] have suggested that low MW chitosan possesses high antioxidant activity and antilipidemic effect, while high MW chitosan significantly reduces the levels of pro-oxidants such as LDL in the gastrointestinal tract in a metabolic syndrome rat model. Many studies have shown that chitosan can prevent the accumulation of liver lipids and enhance fecal lipid excretion [10,20]. In the present study, a high-fructose diet induced an increase in liver lipids accumulation and high MW chitosan supplementation; however, it reversed the increase in hepatic lipids and increased fecal lipid excretion. In addition, chitosan reversed the increased liver acetyl CoA carboxylase and fatty acid synthase activities induced by HF-diet. We have reported that chitosan can suppress downstream expression of lipogenic transcription factors through AMPK activation and lipogenesis-associated genes inhibition, which can attenuate TG and cholesterol accumulation in liver [20]. Therefore, chitosan improves the accumulation of liver lipids induced by the HF-diet; this may be related to the activated AMPK phosphorylation and lipogenesis-associated genes inhibition in an HFD rat model. Further investigation is needed to understand the detailed actions and mechanisms of chitosan on lipid responses and lipid metabolism under an impaired glucose tolerance condition.

## 4. Experimental Section

### 4.1. High-MW Chitosan

High-MW chitosan, which was obtained from crab shell chitin by alkali fusion, was obtained from Taiwan Tanabe Seiyaku Co. (Taipei, Taiwan). The average MW and degree of deacetylation (DD) of chitosan were determined by high-performance liquid chromatography and Fourier transform infrared spectroscopy, respectively. The DD of chitosan was about 89.8% and the average MW and viscosity of chitosan were 3.8 × 105 Dalton and 33 mPa.s., respectively. Fructose powder was purchased from Archer Daniels Midland Co. (Chicago, IL, USA) and cellulose was purchased from Sigma Chemical Co. (St. Louis, MO, USA). The degree of deacetylation of chitosan was about 90%, and the average MW was about 380 kDa.

### 4.2. Animals and Diets

Male seven-week-old Sprague-Dawley (SD) rats were purchased from BioLASCO Taiwan Co., Ltd. (Taipei, Taiwan). Rats were fed a chow diet (Rodent Laboratory Chow, Ralston Purina, St. Louis, MO, USA) for 1one week, and then the animals were randomly divided into three groups: a control group that received a control diet, an HF group that received a high-fructose (63.1%) diet, and a CS group that received the HF diet with 5% chitosan. Each group contained eight rats. The compositions of the experimental diets given to test animals are shown in Table 6. Rats were housed in individual stainless-steel cages in a room kept at 23 ± 1 °C and 60% ± 5% relative humidity with a 12 h light and dark cycle (lighting from 8:00 a.m. to 8:00 p.m.). Food and drinking water were available *ad libitum* and measured daily. The body weight was measured every week. The OGTT (oral glucose tolerance test) and ITT (insulin tolerance test) experiments were performed after 16 and 18 weeks of the feeding study, respectively. For the OGTT experiment, rats in each group orally received the glucose solution (2 g/kg of body weight (BW)) in the fasted state. For the ITT experiment, rats in each group were injected with insulin by i.p. (0.75 IU/kg) in the fasted state. Blood samples were obtained at 0 (before diet loading), 30, 60, and 120 min, from the tail vein, using a heparinized capillary tube. Plasma was isolated immediately by centrifugation at 1570× *g* for 20 min at 4 °C and stored at −80 °C until assay. After the 21 weeks of the feeding study, the animals were sacrificed. This study was approved by the Animal House Management Committee of the National Taiwan Ocean University. The animals were maintained in accordance with the guidelines for the care and use of laboratory animals as issued by the Animal Center of the National Science Council.

**Table 6:** Composition of experimental diets (%).

Ingredient (%)	Normal *	HF	HF + C
Corn starch	63.1		
Fructose		63.1	63.1
Casein	20	20	20
Lard	5	5	5
Soybean oil	1	1	1
Vitamin mixture ^1^	1	1	1
Mineral mixture ^2^	4	4	4
Cholesterol	0.5	0.5	0.5
Cholic acid	0.2	0.2	0.2
Choline chloride	0.2	0.2	0.2
Cellulose	5	5	
Chitosan ^3^			5

* Normal: normal control, HF: high fructose, HF + C: high fructose + chitosan. ^1^ AIN-93 vitamin mixture; ^2^ AIN-93 mineral mixture; ^3^ The average MW and viscosity of chitosan were about 3.8 × 10^5^ Dalton and 33 mPa.s., respectively. DD was about 89.8%.

### 4.3. Collection of Blood and Tissue Samples

At the end of the experimental period, animals fasted for 12 h prior to being sacrificed (at 10:00 a.m.) by exsanguinations via the abdominal aorta while under diethyl ether anesthesia. Heparin was used as the anticoagulant. Plasma was separated from the blood by centrifugation (1750× *g*) at 4 °C for 20 min. The liver and soleus muscle from each animal were excised and weighed.

### 4.4. Determination of Plasma Glucose, Insulin, Adiponectin, Leptin, Free Fatty Acids, and Tumor Necrosis Factor-α (TNF-α)

Plasma glucose was determined with a kit purchased from Audit Diagnostics Co. (Cork, Ireland). Plasma insulin was determined using a rat insulin enzyme-linked immunosorbent assay (ELISA) kit (Mercodia AB, Uppsala, Sweden). Plasma adiponectin, leptin, and TNF-α levels were determined using the rat ELISA kits (Assay Designs, Inc., Ann Arbor, MI, USA). Plasma free fatty acid was determined using a kit purchased from Wako Pure Chemical Co. (Osaka, Japan).

### 4.5. Determination of Plasma Lipid Concentration

Plasma TC, and TG levels were determined by an enzymatic method provided by the kits purchased from Audit Diagnostics (Cork, Ireland). The TG-rich lipoproteins from a separate aliquot of plasma were isolated by density gradient ultracentrifugation (Hitachi, SP 85G, RPL 42T Rotor, Tokyo, Japan) by 194,000× *g* at 10 °C for 3 h, and the lipoproteins were recovered by tube slicing [37].

### 4.6. Determination of Hepatic Lipid Metabolism Enzymes

Hepatic fatty acid synthase (FAS) and acetyl CoA carboxylase activities were assayed as described previously by Goodridge [38] and Nepokroeff *et al.* [39] respectively. The enzyme activities were determined by the rate (nmole/min/mg protein) of NADPH increase.

### 4.7. Lipolysis Rate Measurement

Adipose tissue (0.2 g) was minced into small pieces and placed in 2 mL of 25 mM *N*-tris-(hydroxymethyl)methyl-2-aminoethanesulfonic acid (TES) buffer containing 1 μM isoproterenol and incubated at 37 °C. Isoproterenol produced a dose-dependent increase in lipolysis. There was a time-dependent increase in lipolysis. After 1, 2, and 3 h of incubation, 0.2 mL of medium was used to measure the levels of glycerol by a commercial reagent (RANDOX GY105, Amtrim, UK), and then the absorbance at 405 nm was recorded using a spectrophotometer. The lipolysis rate was indicated by micromoles of glycerol released per gram of tissue per hour [40].

### 4.8. Western Blot Analysis

Total protein containing 50–100 μg was separated on 8% sodium dodecyl sulfate (SDS)-polyacrylamide minigels and transferred to nitrocellulose membranes (GE Healthcare, Marlborough, USA). After blocking, blots were incubated with antibodies for α-tubulin, angiopoietin-like 4 (ANGPTL4) (Santa Cruz, Dallas, USA), glucose transporter-4 (GLUT4) in phosphate-buffered saline (PBS)/Tween-20 for 1 h, followed by two washes in PBS/Tween-20, and then incubated with horseradish-peroxidase-conjugated goat anti-mouse IgG for 30 min. Moreover, α-tubulin served as a control for sample loading and integrity. The antibody-reactive bands were revealed by the enhanced chemiluminescence kit (GE Healthcare, Marlborough, USA) and were used to expose to Kodak radiographic film. The amount of polypeptide was quantitated by integrated densitometric analysis of the film (Kodak Gel Logic-100 Imaging System, Rochester, USA).

### 4.9. Statistical Evaluation

Results are given as the mean ± standard deviation (SD). Statistical differences among groups were calculated by analysis of variance (ANOVA) (SAS Institute, Cary, NC, USA), and group means were considered to be significantly different at *p* < 0.05 as determined by Duncan’s multiple range test.

## 5. Conclusions

In this study, we demonstrate that supplementation of chitosan in the diet can effectively mitigate the increased body weight, hypercholesterolemia, and increased insulin resistance induced by HF-diet feeding in rats. These findings suggest that chitosan possesses the ability to improve the impairment of glucose and lipid metabolism in a HF-diet-fed rat model.
